# Sub-Band Spectrum Engineering via Structural Order in Tapered Nanowires

**DOI:** 10.1021/acs.nanolett.1c03071

**Published:** 2021-12-09

**Authors:** Man Suk Song, Tom Koren, Magdalena Załuska-Kotur, Ryszard Buczko, Nurit Avraham, Perla Kacman, Hadas Shtrikman, Haim Beidenkopf

**Affiliations:** † Department of Condensed Matter Physics, Weizmann Institute of Science, Rehovot 7610001, Israel; ‡ Institute of Physics, Polish Academy of Sciences, Aleja Lotnikow 32/46, Warsaw PL-02-668, Poland

**Keywords:** MBE, InAs, kink, nanoflag, tapered nanowires, STM, Monte Carlo simulation, Majoranas

## Abstract

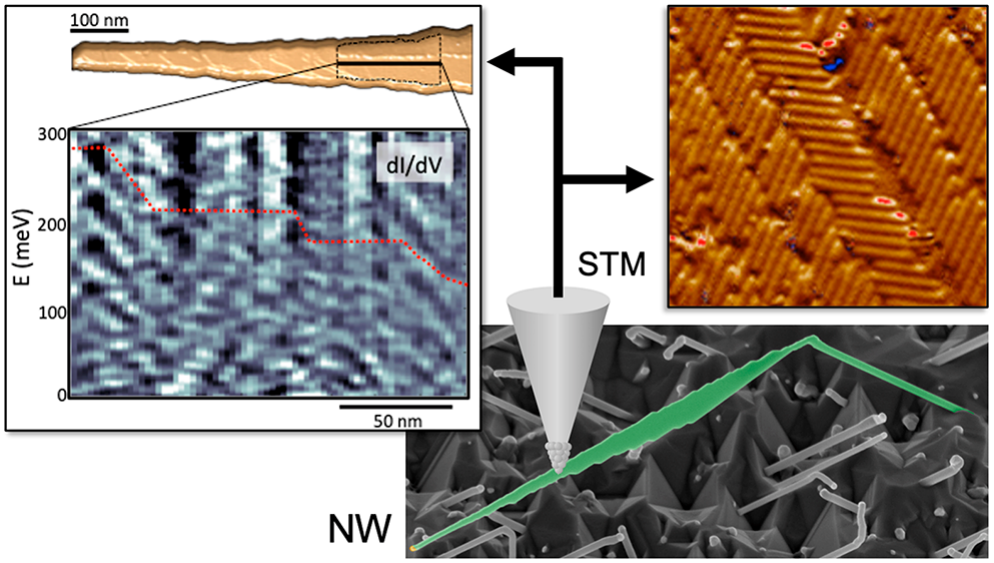

The cross-sectional dimensions of nanowires set the quantization conditions for the electronic subbands they host. These can be used as a platform to realize one-dimesional topological superconductivity. Here we develop a protocol that forces such nanowires to kink and change their growth direction. Consequently, a thin rectangular nanoplate is formed, which gradually converges into a very thin square tip. We characterize the resulting tapered nanowires structurally and spectroscopically by scanning and transmission electron microscopy and scanning tunneling microscopy and spectroscopy and model their growth. A unique structure composed of ordered rows of atoms on the (110) facet of the nanoflag is further revealed by atomically resolved topography and modeled by simulations. We discuss possible advantages tapered InAs nanowires offer for Majorana zero-mode realization and manipulation.

## 1 Introduction

Majorana zero modes (MZMs) at the ends of one-dimensional (1D) topological superconductors are expected to exhibit nontrivial braiding statistics,^1,2^ opening a path toward topologically protected quantum computing.^3,4^ Among the proposals to realize such MZMs, an approach based on semiconducting nanowires (NWs) with strong spin–orbit coupling subject to a Zeeman field and the superconducting proximity effect has received particular attention. The size of the NWs quantizes their electronic spectrum into quasi-1D sub-bands. Strong spin–orbit coupling, such as that found in InAs and InSb NWs,^5−8^ lifts their spin degeneracy except at the Kramer’s degeneracy, which is protected by time-reversal symmetry. The removal of the remaining degeneracy by an external magnetic field induces a Zeeman gap within which the sub-bands spectra are approximately helical. It was predicted that inducing superconductivity within such a helical mode would give rise to a 1D topological superconducting state. This exotic state would harbor MZMs at its ends, which would be localized either at the physical ends of the NW or at the ends of the superconducting electrode needed to induce it by proximity. Indeed, zero bias conductance peaks have been measured both in InAs and in InSb.^5−8^

For this to occur, the superconducting gap induced at the chemical potential and the Zeeman gap induced at the time reversal symmetric points must overlap. This places a strict constraint on the relative energy separation between the chemical potential and the Kramer’s degeneracy points in the spectrum as well as their homogeneity along the NW. While the former is commonly tuned by capacitive gating, the latter has proven to be a challenge. Nevertheless, the mature field of NW research offers a diverse toolbox of growth protocols, geometries, and phenomena that can be harnessed to improve the properties and robustness of the electronic spectra. It enables, for instance, the careful deposition of superconducting electrodes and the engineering of their interface with the semiconducting NW and its electronic transparency. Such core–shell structures were used recently to produce an orbital variant of the topological superconducting state.^9^

Another important challenge is the production of devices suitable for performing nonabelian braiding operations. This has driven the quest for the formation of NW intersections and their networks as the “Y” trijunctions and “X” (or “K”) crosses over which MZMs can be interchanged and their predicted nonabelian statistics can be tested.^2^ We note that III–V semiconducting NWs of a zinc blende (ZB) structure grow preferentially along the ⟨111⟩ orientation or the equivalent ⟨0001⟩ direction in the case of wurtzite (WZ) NWs. Hence, kinking or bending of the NWs has been used to form basic intersections among NWs that grow normal to a (111)-oriented substrate.^10^ Since NWs commonly grow along the ⟨111⟩ axis regardless of the substrate orientation, NWs growing on substrates other than (111)-oriented substrates typically grow inclined to the substrate surface. Merging inclined NWs into intersections and more complex networks is much more straightforward.^11−13^ Moreover, it was shown that the tilt angle assists the side coating of the NWs and intersections by a superconducting metal.^13^ This allows the even coverage of multiple arms of the coated NW intersection.^13,14^ On the (001) substrate, inclined InAs NWs can emerge only in two different ⟨111⟩ directions, making this substrate particularly suitable for the formation of regular NW networks. This is due to faceting by the Au-induced formation of microcraters with two mirror-symmetric opposite {111}B side facets.^15^

Apart from these achievements, many of the advantages of NW toward supporting a robust platform for the realization and manipulation of Majorana modes have not been explored yet. Here, we investigate the growth dynamics, crystallographic structure, and spectroscopy of tapered NWs in which the diameter evolves along the NW axis. The gradually varying confinement of electrons in the NW along its axis modifies the electronic spectrum. Such technology may allow NW segments to be autotuned into their topological superconducting state as well as the transportation of those segments along the tapered NW. Calculations even predict enhanced topological protection in planar quasi-1D channels with a periodically modulated width.^16^

## 2 Tapered NW Morphology and Structure

**Figure 1:**
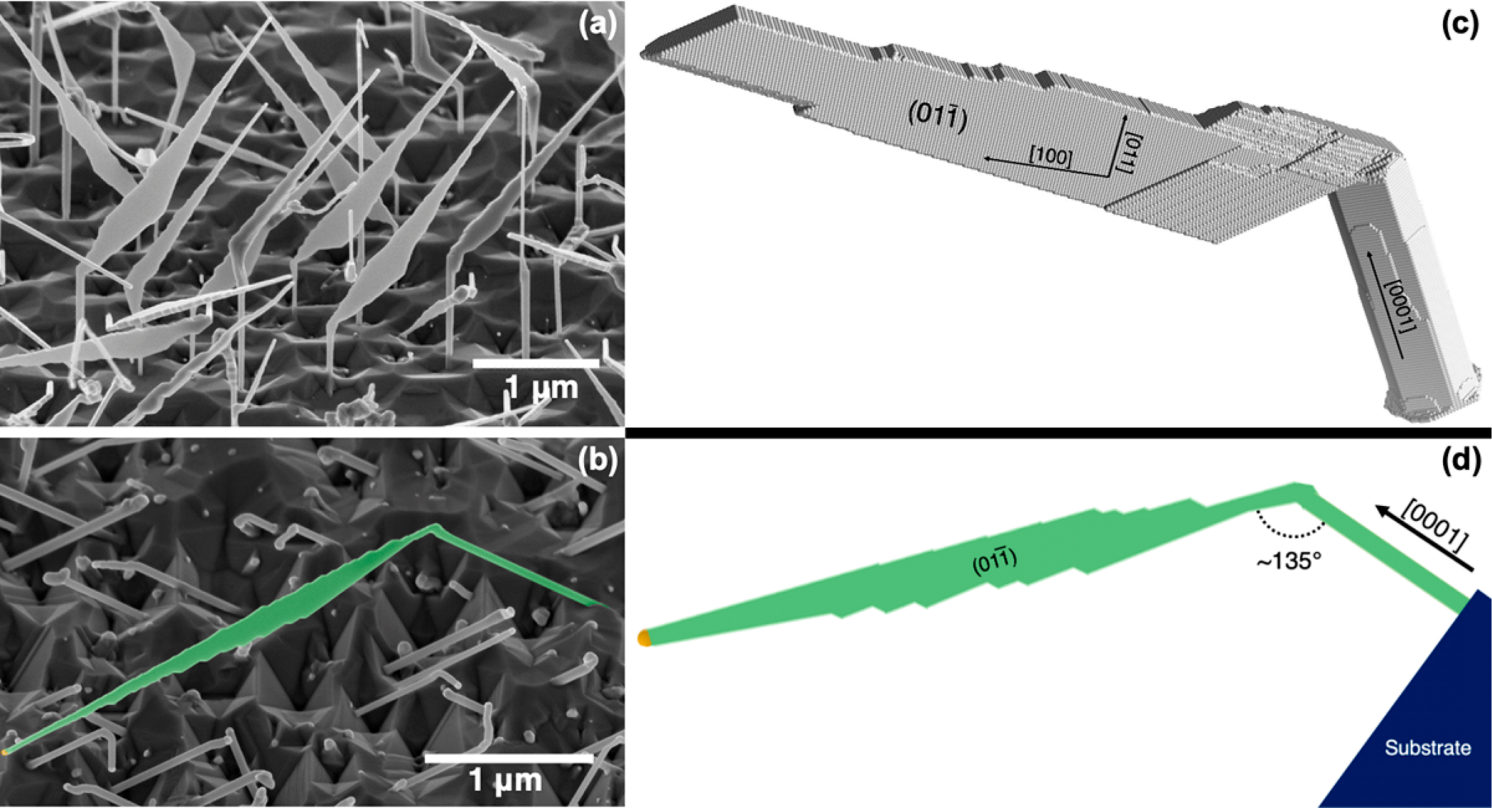
Kinked InAs NWs. As-grown kinked InAs NWs emerging from an InAs (001) faceted surface. (a and b) Their irregular and elongated shape is clear from the scanning electron microscope (SEM) images. (c) The shape of the kinking NW modeled by Monte Carlo simulations. (d) A schematic illustration of the nanoflag NW.

Kinked InAs NWs were grown by Au-assisted vapor–liquid–solid (VLS) molecular beam epitaxy (MBE) on the (001) plane, which produced rounded reclining NWs that emerged in two opposite ⟨111⟩ directions (see Figures 1a and b and S1). By lowering the growth conditions (by 100 °C in this study), such NWs are forced to kink and change their growth direction, structure, and shape.^17^ The kinking of the stem growing in the ⟨111⟩ direction into the new growth direction induces a significant change in the NW morphology. They bend into the direction perpendicular to the [011] axis, i.e., a ⟨*mnn*⟩ direction with *m* ≫ *n*, assuming a change from the WZ structure to the ZB one.

The rectangular nanoplate that forms after the kink is characterized by two narrow facets (about 40 nm thick) and two broad [011̅] facets, as shown in Figure 1 (see also Figure S1). The edges of the two broad facets are terminated by prominent macro steps of inclined (111) facets. The broad facets gradually converge into a very narrow square-shaped tip (about 30 nm in both thickness and width). During the growth, the Au droplets shrink into significantly smaller droplets beyond the kink. This is a result of the temperature decrease, which increases the supersaturation in the droplets. The kinking does not take place in any particular direction (Figure S2a). The necessary condition for kinking the InAs NWs is lowering growth temperature (100 °C in this work). It is worth mentioning that the smaller the NW’s diameter, the higher the temperature at which kinking will occur.

**Figure 2:**
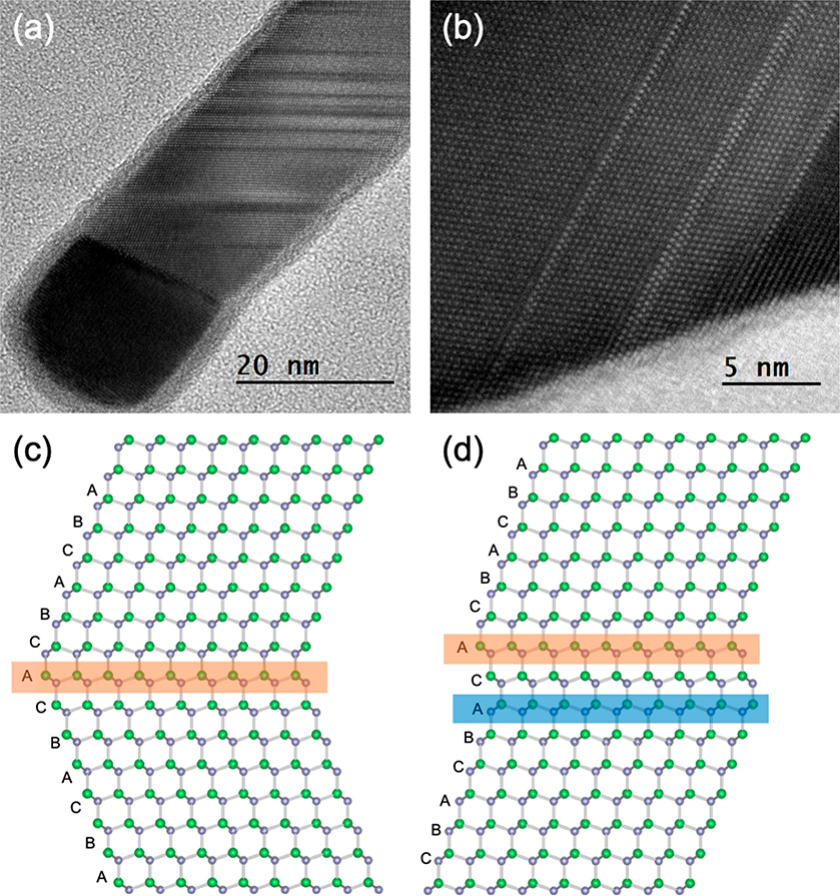
Twin plane in tapered NWs. (a) TEM image showing the tip of a kinked NW with a ZB structure and occasional diagonal double twin planes. (b) High-resolution TEM image showing more clearly a couple of diagonal double twin planes. Schematic illustrations of (c) a single and (d) a double twin plane.

A broad neck with occasional diagonal double twin planes forms between the WZ stem and the ZB nanoplate (SI, Figure.S3). The double twin planes are visible in high-resolution transmission electron microscopy (TEM) images, as shown in Figure 2a and b. Schematic illustrations of a single and a double twin plane are shown in Figure 2c and d, respectively, which clarify the atomic layers ordering.

These NWs resemble the so-called nanoflags.^18^ Occasional crosses form by the intersection of two nanoflags (Figure S2b). Such an intersection could be a skeleton for the formation of a device that can display the braiding of Majoranas (Figure S2c), as discussd below.

## 3 Simulation of Tapered NW Growth

To explain the shape and structure of the kinked InAs NWs, their growth was simulated using the MC method. Our simulations start by assuming an external flux of particles approaching the surface with given frequency. As the VLS growth of the InAs NWs in MBE is conducted at a high As overpressure, we assume indium is the element that fully controls the growth. Thus, in the calculations the external flux consists of only one type of particle, i.e., indium atoms. The In adatoms diffuse along the surface and can either nucleate, creating clusters, or attach to steps that exist on the surface. Each of these processes is governed by a different probability. An increased probability for forming clusters is assumed on a 20 lattice units wide part of the surface to simulate the presence of a gold droplet, which catalyzes the NW growth. It is also assumed that the diffusing particles can easily climb up a step on the surface but that coming back is forbidden. In such a way we model the process of capturing adatoms on the part of the surface simulating the gold spot. NW growth starts from the nucleation of a seed consisting of four neighboring In atoms on the flat surface within the gold droplet. Each new layer on top of the growing NW also starts with a seed of nucleation. As we have shown already in ref (15), such a modeling scheme allows us to nicely simulate the process of Au-assisted NW growth.

In this study, we first simulate the growth of a NW vertical to the (111) surface, forming a WZ structure. The attachment of adatoms on the hexagonal top of the NW is equally probable in all six directions, with the rate of 0.09. In the simulation, this NW was grown along the [0001] axis for 33 000 MC steps. As a result, a 180 lattice units high regular hexagonal NW was obtained. Next we bend the NW, following the reasoning presented in ref (17) that lowering the temperature changes the balance between the free energy of the gold droplet and the chemical potentials of different surfaces. Thus, the gold droplet can move to the side of the NW. The new NW beneath such gold spot would grow in a different direction with respect to the original one. To include this process into the calculations, we first move the area that simulates the gold droplet to the side of the NW and rotate the whole structure to have the vertical axis in the [311] direction. Then, performing the growth process upward, we obtain a kinked NW pointing in a direction laying between the [311] and [100] axes (Figure 1c). At the same time, the geometry of the new surface induces a new crystal structure, namely ZB. We model the new structure by assuming a diffusion coefficient twice as small as that in the first stage. It is in line with the reduction of temperature, which causes the NW bending in the experiment. The lower growth temperature and slower diffusion increase the nucleation probability as well as the particle’s likelihood to attach to a step. Moreover, to obtain a flat rectangular NW with wide (110) facets, we assume a 10^3^ larger attachment probability in the [100] than that in the [110] direction. The diffusion of adatoms along the surface sets the time scale of the simulation. Thus, this part of the simulations was run for 300 000 MC steps, nine times longer than the first one.

## 4 Spectroscopic Characterization of Tapered NWs

**Figure 3:**
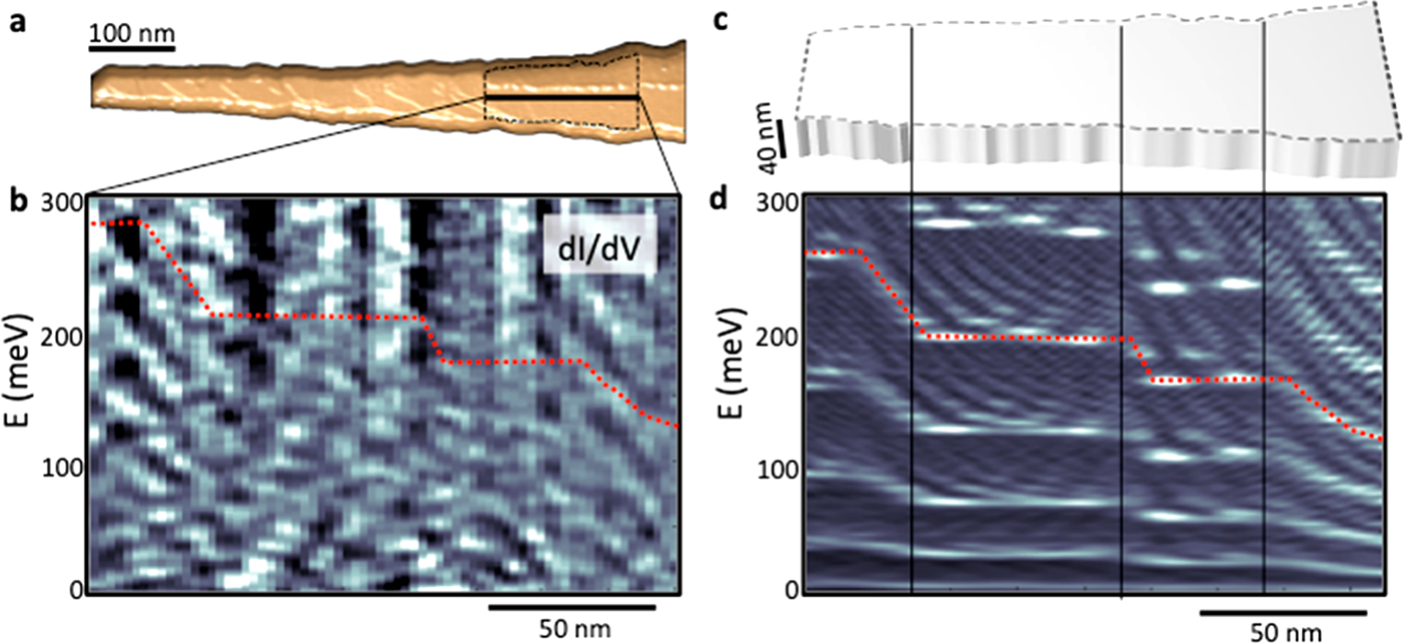
Density-of-state response to tapering. (a) Topographic STM image of a segment of a tapered NW. (b) d*I*/d*V* measured along a line displaying the spectrum evolution. (c) Modeled segment of a NW based on the topography in panel a. (d) Kwant simulation of local density of states within the tapered NW in panel c. Dotted lines in panes b and d are guides to the eye.

The obtained rectangular cross-section of the nanoflag with its flat facets renders the kinked NWs highly suitable for scanning tunneling microscopy (STM) studies. We harvested the NWs onto a Au substrate and transferred them in a designated ultrahigh vacuum suitcase, as developed in our previous studies.^19,20^ The results of the STM measurements showing the topography and demonstrating the impact of tapering on its energy spectrum are presented in Figure 3.

The tapered topography of the NWs is clearly visible in Figure 3a along with its irregularities. A milder thickness decrease was also imaged by the sequence of step edges on the top flat terrace. We map the evolving local density of states over an atomically flat linecut along the NW axis in Figure 3b. The fairly regular bright spots at any point along that line stem from Van Hove singularities at sub-band extrema. The local irregularities of the tapered NW boundaries, as well as the surface roughness on the atomic scale, result in a complex evolution of the spectrum. Nevertheless, along the line scan this rather regular pattern evolves in energy in response to the overall varying boundaries of the tapered NW. These main spatial patterns are reproduced at different positions across the tapered NWs (see Figure S4).

We have modeled the detailed NW boundaries along this segment (Figure 3c) and simulated the density of states response in Kwant (Figure 3d extracted from the topography in Figure S5). We identified four distinct sections in the topography with clear spectral correspondence. In the right and left most sections, the level spacing gradually increases as the NW gradually shrinks. Along the central two sections, the width is fixed but changes abruptly at a certain point, as does the level spacing. Those spectral trends in the simulation (red line in Figure 3d) are exhibited by the tapered NW spectroscopically mapped in Figure 3b. This exemplifies how the quantized spectrum of a NW can be engineered by modulating its boundaries. However, more regular tapering will be required to achieve the gradual and monotonic evolution of the spectrum along the NW axis, which will support the smooth control and transportation of topological superconducting segments along it.

## 5 Atomic-Scale Superstructure in Tapered NWs

**Figure 4:**
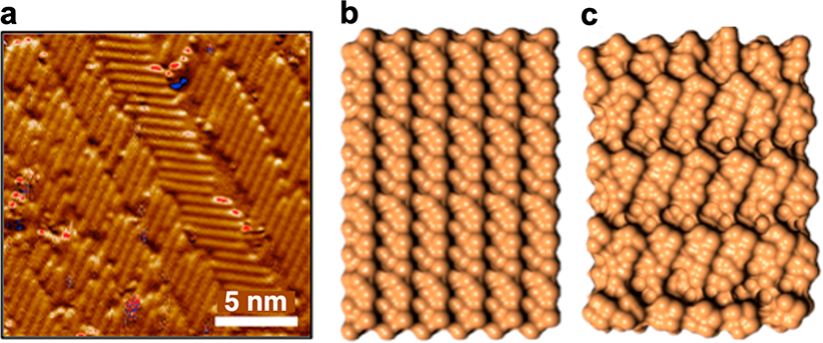
Surface superlattice potential. (a) Representative STM topography of the atomic arrangement of the (110) surface showing 4 unit cell chains arranged in rows. (b) Initial As adatom placement a non-(110) InAs surface (In ions are not visible). (c) As adatom reconstruction into the superstructure following a minimization procedure within the LAMMPS molecular dynamics simulator Tersoff potential transforms.

STM topography further discovered a self-ordered atomic pattern at the surface of the kinked NWs. It consists of four-atom chains that form rather regular rows (Figure 4a). We did not detect a similar pattern in TEM (Figure 2), which is a bulk probe, suggesting it is strictly a surface phenomenon. This pattern resembles the surface reconstruction reported for differently oriented surfaces in several III–V compounds in the presence of excessive ions, either cations^21,22^ or anions.^23^ Thus, as the MBE growth of our NWs is conducted under As overpressure, we assert that the reconstruction pattern observed in the STM relates to the excess As ions at the surface. We note that As termination of the (110) surface of GaAs indeed leads to a lower energy than the cationic one.^24,25^

To test the hypothesis that the patterns at the InAs (110) surface of the nanoflags are related to As adatoms, we performed a minimization procedure within the LAMMPS molecular dynamics simulator Tersoff potential.^26^ The parametrization for the InAs crystals was taken from ref (27). For this procedure, a crystal composed of 12 × 12 × 8 atomic layers with a ZB structure and atomic distances typical of InAs was arranged. The top surface was a (110) plane, over which we placed 144 As adatoms in regular rows. The number of additional As atoms is equal to the number of atoms in one monolayer. The initial arrangement is shown in Figure 4b, where only As ions, denoted by yellow balls, are visible. Periodic boundary conditions were set in the *x*- and *y*-directions, while in the *z*-direction the boundary was left free. Two layers at the bottom of the crystals were kept immobile, whereas a large space of three interlayer distances was left above the crystal surface. This distance allows atoms to move freely on top of the surface. The crystal structure was relaxed using a conjugate gradient algorithm. Figure 4c shows the configuration after relaxation. After such a reconstruction, As atoms form regular chains that are inclined to the initial rows. The consecutive chains of As atoms are well separated from one another. Like in the STM picture shown in Figure 4a, the pattern breaks periodically along [100] lines. At each such break, the whole next chain setup is shifted. The breaking lines, which appear both in STM pictures and in the simulations, are formed in order to relax the strain arising from the mismatch between the InAs (110) surface and the As chains.

In Figure 4a, one can also notice a change of the orientation of the As chains at some line that is not visible in the simulations, suggesting that the orientation changes of the surface pattern originate in the bulk ordering beneath the surface. Indeed, the TEM images (Figure 2) confirm that the structure we observe in the STM images is present only at the surface, whereas below it a very uniform crystal structure is visible. Moreover, we can identify the plains along which the surface pattern changes its orientation, originating in the bulk ordering. Finally, it should be noted that the angle between the two orientations of the chains indicates that the real crystallographic directions of the As chains are either the {211} type or the {111} type . Unfortunately, our simulations do not reproduce the direction of the chains, as the twist is too small and the simulated chains are oriented along the [322] axis. This can be improved by a better choice of parameters. Yet, the present results confirm that the pattern observed on the (110) surface of the nanoflags is indeed related to the As adatoms.

Remarkably, a 4 unit cell potential induces the Brillouin zone folding needed to have a Kramer’s degeneracy right at the vicinity of the chemical potential in InAs NWs.^28^ Hence, its presence may fully alleviate the need to further tune the chemical potential or will at least substantially reduce the amount of tuning needed. The combination of superlattice folding with mild tapering may be ideal to maximize the benefit of both, as mentioned in section 1. Further research and development of this concept are needed to distill these effects from other crystallographic irregularities.

## 6 Discussion

**Figure 5:**
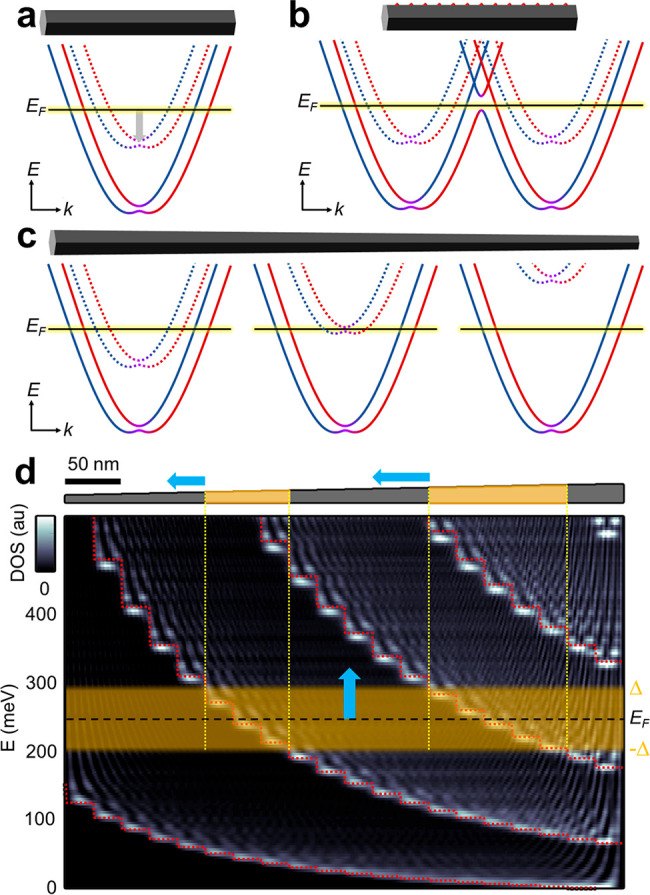
Approaches to tuning Kramer’s degeneracy to the chemical potential and to manipulating Majorana modes. (a) Typically, the chemical potential is tuned by capacitive gating. (b) The periodic atomic-scale potential will fold the quantized band structure, inducing additional Kramer’s degeneracies at the edges of the folded Brillouin zone that can be tuned to the chemical potential by engineering the periodic potential. (c) Tapered NWs, where the diameter varies along the NW, will vary the sub-band energy gaps with pushing Kramer’s degeneracies across the chemical potential. (d) The Van Hove singularities seen in the Kwant simulation (false color) of the tapered NW (top panel) follow a naïve quantum particle in a box calculation (dotted line). Inducing superconductivity (yellow shaded) at a certain chemical potential (dashed line) will give rise to topological superconducting segments. Tuning the chemical potential by the gate will transport the topological segments in the tapered NW (arrows).

In the present work, we realize and examine tapered NWs that also host an atomic-scale superstructure on their surfaces. Intriguingly, these may provide two complementary methods for engineering the Kramer’s degeneracy within the vicinity of the chemical potential. First, a periodic superlattice potential folds the sub-band spectrum. This gives rise to additional Kramer’s degeneracies at the edges of the folded Brillouin zone (Figure 5b). An atomic periodicity of four was calculated to be optimal for inducing such Kramer degeneracies in proximity to the chemical potential.^28^ Second, as the NW diameter gradually changes along its axis, it consequently varies the sub-band level spacing. This pushes Kramer’s degeneracies across the chemical potential at certain segments along the NW (Figure 5c). Both methods alleviate or minimize the need for back-gate tuning to induce the topological superconducting phase, as they bring Kramer’s degeneracy close to the chemical potential. Moreover, for the second approach the application of a uniform back-gate would result in a smooth variation of the segments, along which the superconducting gap would overlap with the Zeeman gap. This would result in transportation of the topological segment along the NW (Figure 5d), allowing an unprecedented level of control over the MZMs. Tapered NW crosses may even provide a route to perform braiding operations with the minimal required number of gates (see Figure S2), thus reducing the complexity and improving the scalability of Majorana networks. Remarkably, we find that both these approaches have the potential of being realized in tapered NWs. Yet, the improved quality of either the periodicity of the superstructure or the smooth evolution of the tapering is needed before these can be readily demonstrated in tapered topological NWs.

## 7 Conclusions

Tapered, so-called nanoflag, InAs NWs that host an atomic-scale superstructure on their surfaces are presented. We have studied their growth and electronic structure. InAs NWs, which nucleate on a (001) surface with a pure WZ structure, are forced to diverge from the [0001] direction by experiencing low temperature and high supersaturation. The new growth direction induces a change from WZ structure with a typically rounded shape to a ZB tapered rectangular nanoflag with two broad (011) facets. This rectagular shape enables careful STM measurements of the (011) surface of the nanoflag. Studies using SEM, TEM, and STM were correlated and supported by kinetic MC simulations, shedding light on the unique surface structures composed of ordered rows of atoms. In the tapered NWs, the quantized spectrum evolves with the varying NW diameter. The tapered global structure and the microscopic superstructure may provide two complementary methods for engineering the Kramer’s degeneracy to within the vicinity of the chemical potential. Thus, in agreement with recent theoretical predictions, we propose that the nanoflag InAs NW structures should be very well suited for the search and manipulation of MZMs.

## Supporting Information Available

The details of growing kinked InAs NWs, more SEM and TEM images of such NWs, a schematic illustration of the braiding process, additional spectroscopic linecuts across a tapered NW, and a topography image displaying the procedure with which the profile was extracted (PDF)
