# Supplementary material for: Sub-Band Spectrum Engineering via Structural Order in Tapered Nanowires
Source: Nano Lett. 2021 Dec 9;21(24):10215–21. doi: 10.1021/acs.nanolett.1c03071 (PMC8704197; doi:10.1021/acs.nanolett.1c03071)
Supplement: Supplementary file 1 — nl1c03071_si_001.pdf [file nl1c03071_si_001.pdf]

Supplementary Information

Sub-band spectrum engineering via structural order in tapered nanowires

Man Suk Song,[1] Tom Koren,[1] Magdalena Załuska-Kotur,[2] Ryszard Buczko,[2] Nurit Avraham,[1]

Perla Kacman,[2] Hadas Shtrikman,[1] and Haim Beidenkopf[1]*

[1]Department of Condensed Matter Physics, Weizmann Institute of Science, Rehovot 7610001, Israel

[2]Institute of Physics, Polish Academy of Sciences, Aleja Lotnikow 32/46, Warsaw PL-02-668, Poland

1. Method for growing InAs kinked NWs

The high purity InAs kinked NWs were grown by Au-assisted vapor liquid solid (VLS) molecular beam epitaxy (MBE) in a Riber 32 system with vacuum in the low 10^{-11} Torr. A very thin (< 1 nm) layer of Au was evaporated in-situ on the (001) InAs at ~100 ˚C right after oxide blow-off in a separate chamber attached to the MBE growth chamber. For the general NWs growth on the (001) the substrate was first heated to ~600 ˚C under arsenic overpressure (As/In ~ 100), where the gold droplets form, then gradually cooled to the growth temperature ~400 ˚C. Midway between the two temperatures the In shutter (~5e-7) was opened [1]. During this cool down process the (001) surface initially becomes covered with craters comprised of two opposite (111) facets, which facilitate the nucleation of typically rounded NWs that grow in two opposite <111> directions. NWs growth in the <111> direction is maintained for an hour after which the substrate temperature is reduced by 100 ˚C at a rate of 10 ˚C per minute while growth continues all the way to ~300 ˚C. The low temperature growth continued for 1-2 hours for different samples in order to extend the "plate" length.

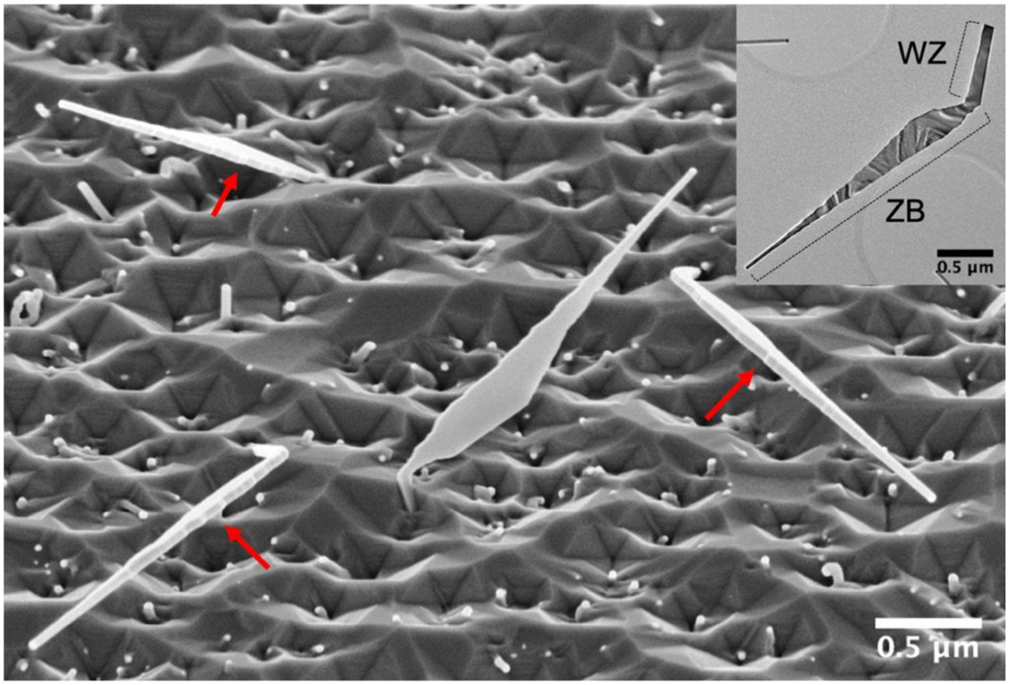

Figure S1: Birds eye view SEM image of low-density InAs nanoflags. The red arrows indicate the edge steps of nanoflag side as compared to the flat 110 surface of the nanoflag seen in the center of the image. The inset shows a low-magnification TEM image of stem (WZ) and kinked and tapered nanoflag (ZB).

2. Braiding in tapered NW crosses

Since the nanoflag NWs orient in various directions (Fig. S2a) occasionally crosses of such tapered NWs occur, as the one in imaged Fig. S2b. Such tapered NW crosses may support a simple and scalable protocol for braiding Majorana zero modes across them. It relies on two main ingredients: the first is the smooth transportation of Majorana modes by gating, which is dictated by the local diameter. The second is that the Zeeman gap is not contributed by the magnetic field component perpendicular to the nanowire and parallel to the substrate. Therefore, for perpendicularly crossing NWs rotation of an in plane magnetic field would make the Zeeman gap in one arm grow while the gap in the other would shrink. Consequently, the rotation results in an enlarged topological segment on one arm and a shrinking segment on the other, respectively. Combination of both

enables the following braiding sequence, sketched in Fig. S2c: at stage (i) both topological segments are removed from the cross. At stage (ii) application of a backgate transports both segments towards the cross so that one segment crosses it while the second does not (dictated by the local tapering). At stage (iii) rotation of the in-plane magnetic field enlarges the segment outside the cross till it eventually crosses the intersection. At stage (iv) the backgate is reversed such that the topological segment that entered first exits the intersection. At stage (v) the magnetic field is rotated back such that the system returns to stage (i) up to an exchange of the Majorana modes that has occurred. This constitutes a braiding operation that requires a single gate per a crossed tapered NWs intersection. For a network of tapered NWs the application of the local gates can be considered as activation of the qubits to be braided, while the rotation of a global in-plane field performs the braiding for those gate activated ones. Those qubits that are not activated are spectators that will not undergo braiding by the rotation of the field.

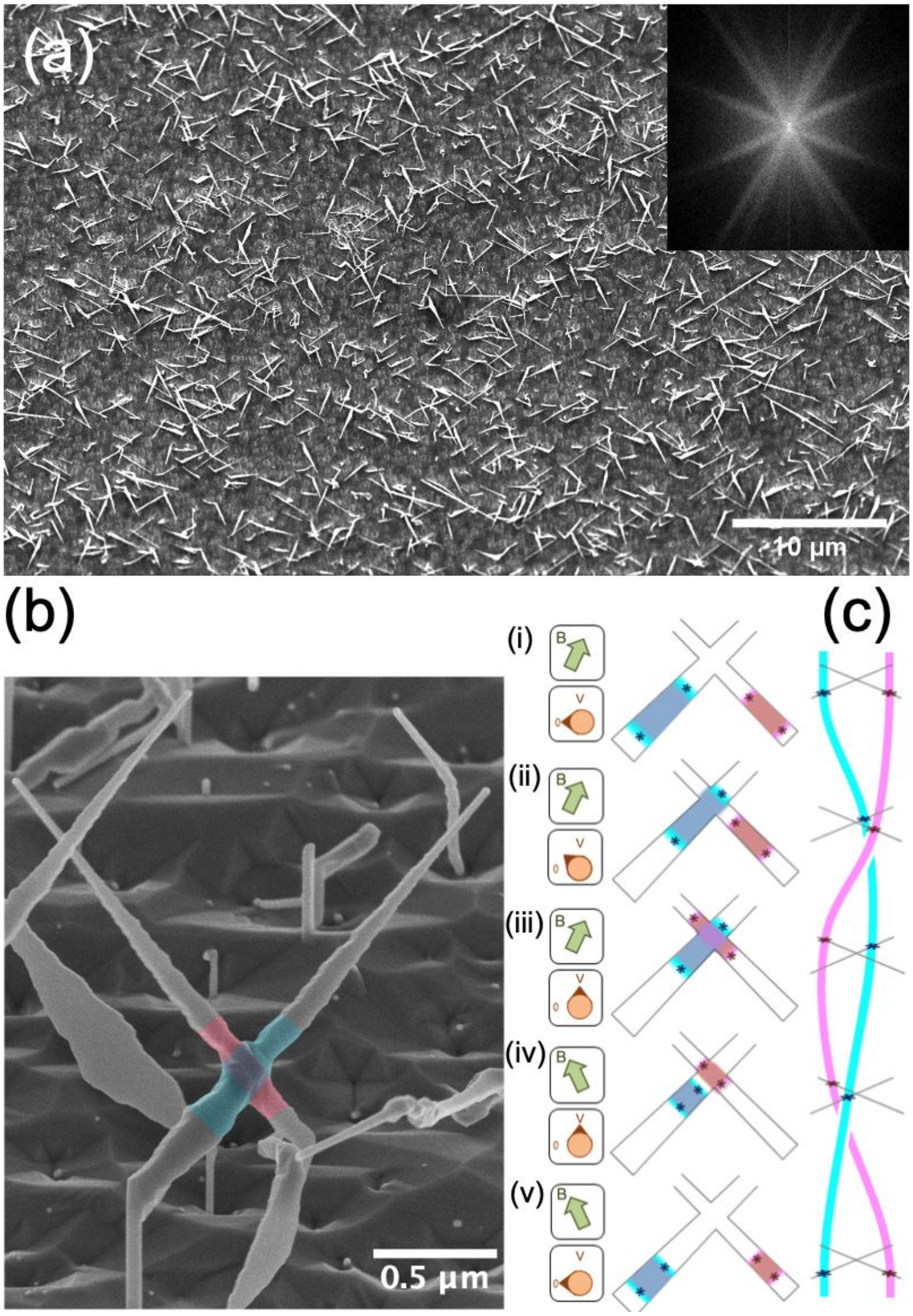

Figure S2: **(a)** Top-view SEM image of an InAs nanoflags sample. Kinked tips point out in particular directions perpendicular to the symmetric lines seen clearly in the relative Fast Fourier Transform (FFT) image (inset). **(b)** Larger magnification SEM image shows that occasionally, two kinked NWs form intersections (as pointed by the red arrow). **(c)** Schematic illustration of braiding of Majorana end modes in a tapered NW cross using a single global back-gate. Rotation of in-plane magnetic field changes the extent of the topological segment (its component along the NW axis does not contribute to the Zeeman gap) and variation of the chemical potential transports the topological segment. The central column follows the positions of the two topological segments according to the magnetic field and potential shown in the left hand column. The right hand column follows the relative position of the four end Majoranas showing braiding of a pair.

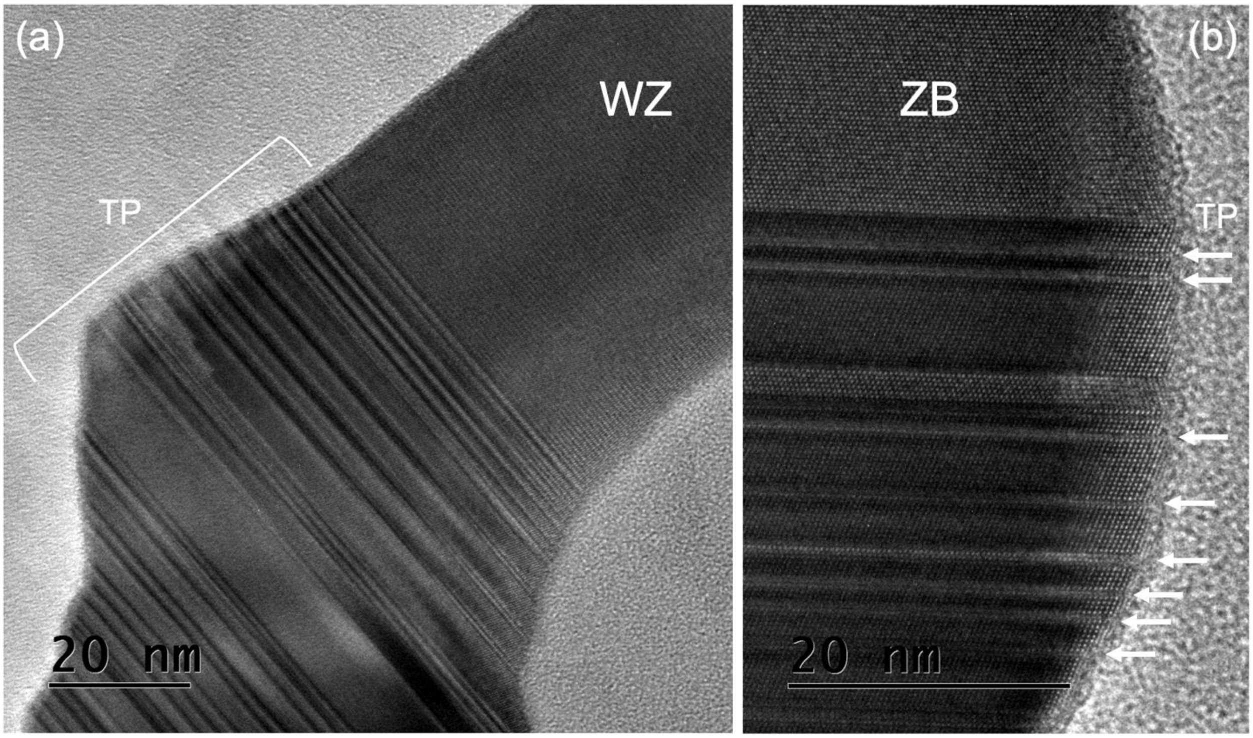

Figure S3: **(a)** TEM images of the kink of the NW showing the change from WZ to ZB structure and **(b)** very unique and typical double twin planes which result from two subsequent rotations of the lattice.

3. Spectroscopic Characterization of Tapered NWs

Figure S4: Two spectroscopic linecuts taken at slightly distant locations across the tapered NW show similar gross features. The top linecut is cropped from the full linecut shown in main text (Fig.3). The bottom one was taken about 5 nm below it over the same distance of 100 nm. Their similarity signifies that the dispersing features result from the overall confinement while local disorder changes only fine details.

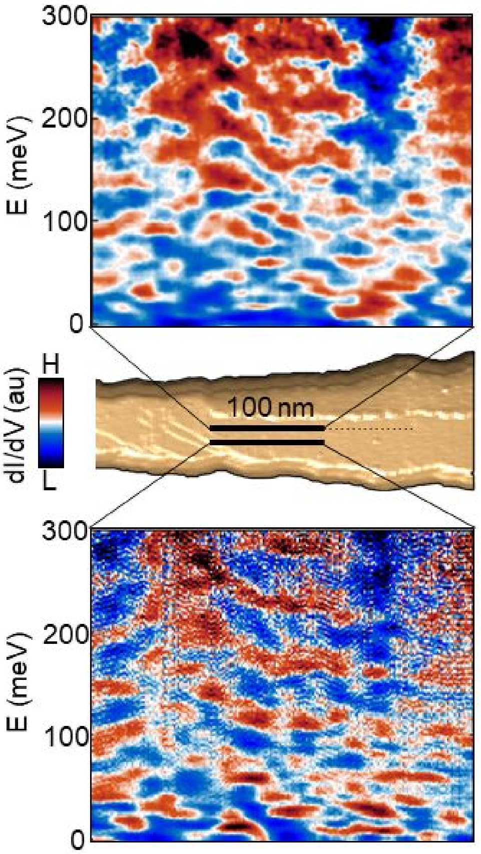

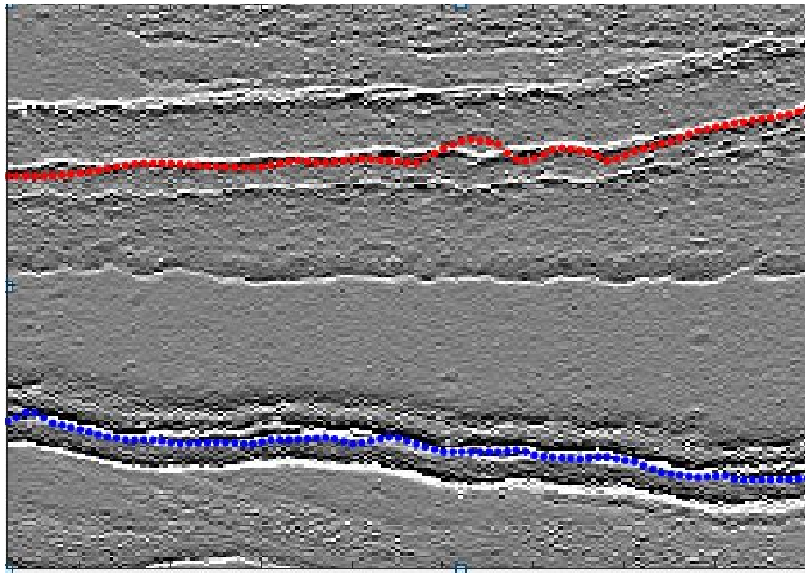

Figure S5: The nanowire profile was extracted for Kwant simulation by following the sharp peaks in the spatial derivative of the NW's topographic image. 100 nm of straight segments of matching width where padded on the two ends to remove artifacts of finite length quantization.

REFERENCES

(1) Kang, J.-H.; Krizek, F.; Zaluska-Kotur, M.; Krogstrup, P.; Kacman, P.; Beidenkopf, H.; Shtrikman, H. Au-Assisted Substrate-Faceting for Inclined Nanowire Growth. *Nano Lett.* **2018**, *18* (7), 4115–4122. https://doi.org/10.1021/acs.nanolett.8b00853.
